# *In vivo* and *in vitro* function of human UDP-galactose 4′-epimerase variants

**DOI:** 10.1016/j.biochi.2011.06.009

**Published:** 2011-10

**Authors:** Thomas J. McCorvie, Jamie Wasilenko, Ying Liu, Judith L. Fridovich-Keil, David J. Timson

**Affiliations:** a School of Biological Sciences, Queen’s University Belfast, Medical Biology Centre, 97 Lisburn Road, Belfast BT9 7BL, UK; b Graduate Division of Biological and Biomedical Sciences, Emory University, Atlanta, Georgia, USA; c Department of Human Genetics, Emory University School of Medicine, Atlanta, Georgia, USA

**Keywords:** Type III galactosemia, Yeast model, GALE, Disease-associated mutation, UDP-galactose 4′-epimerase, ANS, 1-anilinonaphthalene-8-sulphonic acid, gal-1P, galactose 1-phosphate, GALE, UDP-galactose 4′-epimerase, GuHCl, guanidine hydrochloride, hGALE, human GALE, MeOH, methanol, RBC, red blood cells

## Abstract

Type III galactosemia results from reduced activity of the enzyme UDP-galactose 4′-epimerase. Five disease-associated alleles (G90E, V94M, D103G, N34S and L183P) and three artificial alleles (Y105C, N268D, and M284K) were tested for their ability to alleviate galactose-induced growth arrest in a *Saccharomyces cerevisiae* strain which lacks endogenous UDP-galactose 4′-epimerase. For all of these alleles, except M284K, the ability to alleviate galactose sensitivity was correlated with the UDP-galactose 4′-epimerase activity detected in cell extracts. The M284K allele, however, was able to substantially alleviate galactose sensitivity, but demonstrated near-zero activity in cell extracts. Recombinant expression of the corresponding protein in *Escherichia coli* resulted in a protein with reduced enzymatic activity and reduced stability towards denaturants *in vitro*. This lack of stability may result from the introduction of an unpaired positive charge into a bundle of three α-helices near the surface of the protein. The disparities between the *in vivo* and *in vitro* data for M284K-hGALE further suggest that there are additional, stabilising factors present in the cell. Taken together, these results reinforce the need for care in the interpretation of *in vitro*, enzymatic diagnostic tests for type III galactosemia.

## Highlights

► GALE activity and ability to compensate a yeast deletion are correlated. ► The correlation does not hold for an artificial allele, M284K. ► M284K was able to compensate *in vivo*, but had near zero activity in cell extracts. ► M284K exhibits reduced *V*_max_ and increased *K*_m_ compared to wild type. ► M284K is highly unstable and probably largely unfolded in dilute solution.

## 1 Introduction

The enzyme UDP-galactose 4′-epimerase (GALE) catalyses the interconversion of UDP-galactose and UDP-glucose. This reaction occurs in the Leloir pathway of galactose metabolism [1]. In mammals, and other higher eukaryotes, the enzyme also catalyses the isomerisation of UDP-*N*-acetylgalactosamine and UDP-*N*-acetylglucosamine and thus also plays a vital role in the synthesis of precursors for glycoprotein and glycolipid synthesis [2]. Mutations in the corresponding gene leading to reduced expression or enzyme activity are associated with the genetic disease epimerase-deficiency, or type III, galactosemia [3–11]. This is the least well understood form of galactosemia, behind type I (galactose 1-phosphate uridylyltransferase deficiency) and type II (galactokinase deficiency) [12–15]. All three forms can result in the development of cataracts during childhood and abnormal levels of Leloir pathway metabolites in the bloodstream or urine. However the occurrence, and severity, of other symptoms vary widely. These are influenced by which gene is mutated, the precise mutation which occurs, other genetic factors that remain poorly understood, and the patient’s environment (especially diet) [16]. In the most severe forms of galactosemia acute damage resulting from dietary exposure to galactose, generally from milk, can be lethal in the neonatal period. Even in the absence of dietary galactose exposure, however, patients with severe forms of galactosemia may experience long-term negative outcomes including cognitive, speech, ovarian, and other complications. In contrast, patients with the mildest forms of galactosemia suffer only from cataracts which may self-resolve upon dietary restriction of galactose, or which can be resolved by surgery, if needed.

Type III galactosemia was previously categorised into a severe (or generalised) and a mild (or peripheral) form [3,4]. Recent work has demonstrated that there are also intermediate forms and that the disease should be considered as a continuum of phenotypes dictated by *GALE* genotype and the level of residual GALE activity present in key tissues, by other genes present in the patient’s genome, and by the patient’s environment [17]. Analysis of variant forms of GALE associated with type III galactosemia showed that there are a number of biochemical causes of the disease. Reduced GALE catalytic turnover was commonly observed, as was decreased structural stability [11,18–20]. In human cells expressing some disease-associated variants of GALE, the protein aggregated in the cytoplasm [21].

Thus, type III galactosemia ostensibly results from loss of GALE expression or from kinetic impairment and/or instability/aggregation of the protein in key tissues, with an apparent inverse relationship between degree of residual activity and severity of outcome. However, there is often a disparity between the level of GALE impairment detected in red blood cells (RBC) and the levels detected in other cell types, including transformed lymphoblasts; clinical severity correlates, roughly, with residual GALE activity detected in cells other than RBC [16]. Prior studies using a yeast model with a doxycycline-regulated allele of the endogenous yeast *GALE* gene, called *GAL10*, demonstrated a clear inverse relationship between the level of Gal10p (GALE) activity detected in cell lysates and galactose sensitivity of the corresponding cultures [22].

Here we extend prior work, expressing five previously-reported patient-derived alleles of human GALE (G90E, V94M, D103G, N34S and L183P [18,23], and also three novel laboratory-generated variants (Y105C, N268D, and M284K) in a *gal10*-null strain of the yeast *Saccharomyces cerevisiae*. Our results demonstrate that seven of these eight variants tested support the previously described inverse relationship between GALE activity and galactose sensitivity [22]. However, one variant (M284K), contradicted this pattern, appearing to be active *in vivo*, but not *in vitro*. Detailed biochemical analyses of this variant revealed possible causes of the *in vitro*/*in vivo* disparity of function, and reinforced the concern that standard *in vitro* enzymatic assays, as performed in many clinical laboratories, may be insufficient to predict fully the *in vivo* function of some variant alleles.

## 2 Materials and methods

### 2.1 Expression of hGALE alleles in yeast

All yeast manipulations were performed according to standard protocols [24] using haploid strains derived from W303 (MATa *ade2-1 his3-11,15 leu 2-3,112 ura3-1 trp1-1 can1-100 RAD5+*), which was the kind gift of Dr. Rodney Rothstein, Columbia University, New York, NY, USA. The plasmids and yeast strains used in this study are listed in Supplementary Tables S1 and S2, respectively. The plasmids were constructed by subcloning each *hGALE* allele into the low copy number (*CEN*) plasmid, pMM33, or the high-copy number (2*μ*) plasmid, pMM195, both of which have been described before [5,18,20]. The Y105C, N268D, and M284K mutations were constructed using error prone PCR of the wild-type *hGALE* sequence and subsequent insertion into vectors. The plasmids in Supplementary Table S1 were transformed into the yeast strain JFy3835, a *gal10-*, *gal80-* haploid strain also derived from W303, and the resulting yeast strains are listed in Supplementary Table S2.

### 2.2 Western blot analysis

Western blot analyses were performed essentially as described elsewhere [25]. Yeast cells were grown at 30 °C and hGALE was detected using a rabbit polyclonal antiserum (EU69), raised against purified hexahistidine-tagged human epimerase protein, at a dilution of 1:40,000 or 1:50,000. As a control for loading, a second antiserum directed against endogenous yeast cyclophilin A [26] was also included at a dilution of 1:240,000 or 1:120,000. Signals were visualised by enhanced chemiluminescence (ECL, Amersham Pharmacia Biotech), using a 1:5000 dilution of a horseradish peroxidase conjugated secondary antibody directed against rabbit Ig (Amersham Pharmacia Biotech), as recommended by the manufacturer.

### 2.3 Enzyme activities from soluble yeast lysates

Soluble yeast protein lysates were prepared as follows: cell pellets from cultures grown in SGE medium deficient in uracil to an OD_600_ of ∼1 were washed with water and resuspended in lysis buffer (20 mM Hepes-KOH, pH 7.5, 1 mM DTT, and 0.3 mg ml^−1^ BSA) plus protease inhibitors (complete mini, Roche). Lysis was carried out by vigorous agitation with 0.5 mm acid-washed glass beads at 4 °C. Lysates were clarified by centrifugation at 13,000 × *g* for 10 min at 4 °C. To remove small metabolites, supernatants were passed through Bio-Spin 30 columns (Bio-Rad) prior to protein quantification. Protein concentration was determined using the Bio-Rad protein reagent, as recommended by the manufacturer, with a BSA standard curve. Samples were stored at −85 °C until use.

GALE activity in yeast lysates was evaluated by monitoring the conversion of UDP-galactose to UDP-glucose, at 37 °C, as previously described [27] with slight modification. In brief, assays were stopped by the addition of 237.5 μl ice cold water and immediately filtered through 0.2 μm nylon micro-spin columns (Corning 8169) to remove particulates before HPLC analysis.

### 2.4 Yeast growth studies

Growth curves were performed as previously described [27] in synthetic medium lacking uracil with 2% glycerol/2% ethanol (SGE-ura). Log phase cells were diluted back to an OD_600_ equal to 0.4 and grown with constant agitation in 96-well plates (NUNC) at 30 °C. OD_600_ measurements were recorded every 2 h using a micro-plate reader (Bio-Tek Instruments, Model EL808).

### 2.5 Studies of galactose 1-phosphate accumulation in yeast

*S. cerevisiae* JFy3835 expressing either wild-type hGALE, no hGALE, or M284K-hGALE from a centromeric plasmid backbone were grown in SGE-ura medium at 28 °C until OD_600_ = 1, then diluted so that OD_600_ = 0.05, and allowed to double to OD_600_ = 0.1. Galactose was then added (*t* = 0 h) to the growth medium to a final concentration of 0 or 0.01%. Cells (10 ml) were collected for intracellular metabolite analysis at *t* = 0 h, 12 h, and 24 h. Extracts were prepared essentially as described previously [27]. In brief, 10 ml of yeast culture was quenched in 20 ml of 60% (v/v) ice-cold methanol (MeOH). Yeast cells were collected by centrifugation at 2000 rpm for 20 min at 4 °C, then transferred to microcentrifuge tubes and washed once with 1 ml of sterile water. Intracellular metabolites were then extracted by vigorous agitation of the cells for 45 min at 4 °C in CHCl_3_:MeOH:H_2_O, 4:2:1 (v/v/v), with a final volume of 875 μl. The aqueous layer was collected after centrifugation at 2000 rpm for 20 min at 4 °C. The remaining organic phase was re-extracted with 125 μl MeOH and 125 μl water. Aqueous layers were combined and dried under vacuum without heat. Finally, dried metabolites were rehydrated with sterile water to a concentration of 5 mg cell dry mass/ml and filtered through 0.2 μm nylon filters (Alltech) before HPLC analysis.

Metabolites were separated and galactose 1-phosphate (gal-1P) quantified using a DX600 HPLC system (Dionex, Sunnyvale, CA) consisting of a Dionex AS50 autosampler, a Dionex GP50 gradient pump, and a Dionex ED50 electrochemical detector, as previously described [27]. In brief, carbohydrates were separated on a CarboPac PA10 column (250 × 4 mm) with an amino-trap (50 × 4 mm) placed before the analysis column and a borate-trap (50 × 4 mm) placed before the injector port to remove trace amounts of borate from the mobile phase buffers. Samples were maintained at 4 °C in the autosampler tray and the chromatography was performed at room temperature. 20 μl of each sample were injected. The following mobile phase buffers were used for separation of carbohydrates: Buffer A, 15 mM NaOH, and Buffer B, 50 mM NaOH/1 M sodium acetate. The flow rate was maintained at 1 ml min^−1^. Buffers were degassed and maintained under a helium atmosphere. Gal-1P was detected using a low salt gradient procedure: Gradient 1: 98% A and 2% B (−10 to 8 min), a linear increase of B to 30% (8–15 min), a linear increase of B to 50% (15–25 min), hold 50% A and 50% B (25–30 min), a linear decrease of B to 2% (30–35 min). Gal-1P was detected by electrical chemical detection using an ED50 detector consisting of a gold electrode and a pH-Ag/AgCl reference electrode for signal detection by integrated amperometry.

### 2.6 Expression and purification of recombinant proteins

Wild-type human GALE was expressed in, and purified from, *Escherichia coli* as previously described [19]. The codon corresponding to Met-284 in the expression vector was altered using the Quik Change protocol [28]. Following verification of the DNA sequence (MWG-Biotech, Ebersburg, Germany), the mutated plasmid was used to direct the expression of M284K-hGALE. The conditions for expression and purification were essentially the same as for the wild-type protein, except that the cells were grown at 24 °C.

The coding sequence for human UDP-glucose dehydrogenase was amplified by PCR from IMAGE clone [29] number 3916854 using primers which incorporated NcoI and XhoI restriction sites at the 5′ and 3′ ends of the sequence, respectively. The PCR product was digested with NcoI and XhoI and inserted into pET21d which had been previously cut with the same enzymes. The sequence of the recombinant expression vector was verified. This plasmid was then transformed into *E. coli* Rosetta (Novagen). Single colonies resulting from this transformation were picked and grown in 5 ml of LB (supplemented with 100 μg ml^−1^ ampicillin and 34 μg ml^−1^ chloroamphenicol), shaking at 37 °C overnight. This culture was then diluted into 1 l of LB (supplemented with 100 μg ml^−1^ ampicillin and 34 μg ml^−1^ chloroamphenicol) and grown shaking at 37 °C until *A*_600nm_ was between 0.6 and 1.0 (typically 3 h). At this point the culture was induced with 2 mM IPTG and grown for a further 3 h. Cells were harvested by centrifugation (4200 × *g* for 20 min), resuspended in buffer R (50 mM Hepes-NaOH, pH 7.5, 150 mM NaCl, 10 %(v/v) glycerol) and frozen at −80 °C until required.

The cell suspensions were thawed and disrupted by sonication on ice (three 30 s pulses of 100 W with 30 s gaps in between for cooling). The extract was centrifuged (20,000 × *g* for 20 min) to remove insoluble material and the supernatant applied to a 1 ml nickel agarose (Sigma, Poole, UK) column. Once this solution had passed through, the column was washed with 20 ml buffer W (as buffer R, expect with 500 mM NaCl) and the protein eluted with three 2 ml washes of buffer E (buffer W supplemented with 250 mM imidazole). Protein containing fractions were identified by SDS-PAGE (Supplementary Fig. S1) and dialysed overnight at 4 °C against buffer D (buffer R supplemented with 2 mM DTT). The protein concentration was estimated by the method of Bradford [30] using bovine serum albumin as a standard. The protein solution was divided into 100–200 μl aliquots and stored frozen at −80 °C.

### 2.7 Measurement of the steady state kinetic parameters for UDP-galactose 4′-epimerase

Enzyme activity was measured by coupling the production of UDP-glucose to the oxidation of this compound by NAD^+^ catalysed by UDP-glucose dehydrogenase [31]. Reactions (150 μl) were set up in triplicate in 96 well plates. Each well contained 10 mM HEPES-NaOH, pH 8.8, 10 mM NAD^+^ and a variable amount of UDP-galactose. Plates were preincubated for 5 min at 37 °C, and then 1.2 μM recombinant human UDP-glucose dehydrogenase was added. To initiate the reaction, wild-type hGALE or M284K-hGALE was added to a final concentration of 5 nM and 1000 nM respectively. All reactions were monitored at 340 nm for 20 min at 37 °C using a Multiskan Spectrum spectrophotometer (Thermo Scientific). Controls containing no hGALE were routinely used and always gave no detectable rate. Initial rates of NADH production were calculated from the linear portions of the progress curves. These rates (*v*) were then plotted against substrate concentration and the data fitted to the following equations using non-linear curve fitting [32] as implemented in the program GraphPad Prism (GraphPad Software, CA, USA). All points were weighted equally.

(1) $$v=V_{\max}\left[\text{UDP}-\text{galactose}\right]/K_{\text{m}}+\left[\text{UDP}-\text{galactose}\right]$$

where *V*_max_ is the maximum, limiting rate and *K*_m_ is the Michaelis constant [33]

(2) $$v=V_{\max}{\left[\text{UDP}-\text{galactose}\right]}^{h}/K_{0.5}^{h}+{\left[\text{UDP}-\text{galactose}\right]}^{h}$$

where *h* is the Hill coefficient and *K*_0.5_ is the concentration of substrate to give a rate equal to half of *V*_max_ [34].

The goodness of fit to these equations was compared using the *F* test and results are reported for the best fit to the data.

### 2.8 Limited proteolysis

Wild-type hGALE or M284K-hGALE (10 μM) was incubated at 37 °C for 5 min with and without 1 mM UDP-galactose. Trypsin (Sigma) was then added to a final concentration of 600 nM and permitted to digest the protein for 30 min. Digestion was then halted by the addition of an equal volume of SDS loading buffer (125 mM tris–HCl, pH 6.8, 4% (w/v) SDS, 20% (v/v) glycerol, 1% (w/v) dithiothreitol, 0.002% (w/v) bromophenol blue) and denaturation at 95 °C. Samples were then analysed by 15 % SDS-PAGE.

### 2.9 ANS unfolding assay

Wild-type hGALE or M284K-hGALE (2.3 μM dissolved in buffer D) in the presence or absence of 50 μM UDP-galactose were mixed with increasing concentrations of GuHCl (0–4 M, 0.5 M increments, dissolved in 10 mM Hepes-NaOH, pH 7.5). Experiments were set up in triplicate in 96 well plates and incubated at 4 °C for 2 h. The extrinsic hydrophobic fluorophore ANS (Sigma) was then added to the samples to a final concentration of 70 μM and samples were left to incubate at 4 °C for a further 1 h. These reactions were set up alongside controls in which protein was omitted. Fluorescence was then detected in a Spectra Max Gemini X (Molecular Devices) fluorimeter (excitation at 370 nm and emission at 480 nm). The mean fluorescence was then plotted against GuHCl concentration.

## 3 Results

### 3.1 Expression of patient and laboratory-generated alleles of hGALE in a null-background strain of yeast

**Fig. 1:**
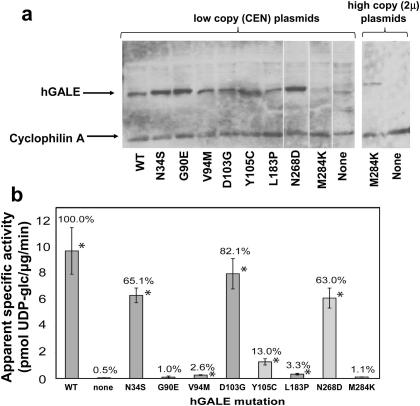
Abundance and activity of hGALE proteins expressed in a null-background strain of yeast. (a) Soluble lysates from yeast expressing the indicated alleles of *hGALE* from low copy number (MM33) or high copy number (MM195) plasmids were subjected to Western blot analysis (see Materials and methods) with antisera specific for hGALE and the endogenous yeast protein cyclophilin A, which served as a loading control. (b) Soluble lysates from yeast expressing the indicated alleles of *hGALE* were assayed for epimerase activity as described in Materials and methods. Values represent the means ± SEM (*n* = 3) of activity normalised to the wild-type control. An asterisk (*) denotes GALE activity that is statistically significant from the negative control using a one-tailed *t*-test (*p* < 0.05).

To test whether differentially impaired alleles of *hGALE* would conform to the inverse relationship previously defined between *GAL10* (yeast *GALE*) expression and galactose-sensitivity in yeast [22] we expressed each of five different patient-derived alleles (N34S, G90E, V94M, D103G, and L183P) and three laboratory-generated alleles (Y105C, N268D, and M284K) in a previously described *gal10*-null strain of *S. cerevisiae*, JFy3835 [35]. Each of the five patient-derived alleles has been demonstrated previously to encode either near-normal, low level, or undetectable levels of GALE activity [18,23], and all three laboratory-generated alleles, which resulted from a random mutagenesis screen for ostensibly temperature-sensitive alleles (see Materials and methods), were novel and previously uncharacterised. Each of these eight *hGALE* alleles was expressed individually from a strong constitutive *GAP* promoter in a centromeric plasmid in the yeast JFy3835, and lysates of the resulting transformants were characterised by Western blot to reveal abundance of the expressed hGALE protein, and by biochemical assay to reveal apparent GALE specific activity level (Fig. 1).

Of the *hGALE* variant alleles tested, all but one (M284K) demonstrated clearly detectable hGALE protein when expressed from a low copy (CEN) plasmid, although there was some variation in abundance (Fig. 1a). Of note, M284K-hGALE protein was detected by Western blot when expressed from a high copy plasmid (Fig. 1a). Given that all of the hGALE proteins were expressed in these transformants from centromeric plasmids bearing the same regulatory sequences, we conclude that the apparent differences in expression level between alleles likely reflect differences in processing or stability of the encoded messages or proteins. Of course, given that our Western blots were performed on soluble cell lysates, we cannot rule out the possibility that during lysate preparation the M284K-hGALE protein became insoluble rather than was degraded.

GALE activity assays performed on soluble lysates from null-background yeast expressing each of the eight variant *hGALE* alleles (see Materials and methods) demonstrated considerable variation in apparent specific activity relative to controls (Fig. 1b). The N34S, D103G, and N268D proteins all demonstrated above 50% residual activity under the conditions tested; the Y105C protein demonstrated just over 10%, and the V94M and L183P proteins each demonstrated less than 5% residual activity. The G90E and M284K proteins each demonstrated no statistically significant GALE activity detectable above the null control.

### 3.2 The anticipated relationship between hGALE function in vitro and in a yeast model system was demonstrated for most but not all alleles

As demonstrated previously (e.g. [18,20]), GALE deficient yeast growth-arrest in the presence of as little as 0.002% galactose, and expression of wild-type human GALE is sufficient to rescue this phenotype. Previously we have also demonstrated that expression of severely impaired patient alleles, such as G90E, V94M or L183P [5,18,23], largely fails to rescue the galactose-sensitivity of otherwise GALE-deficient yeast, while expression of mildly impaired alleles, such as N34S, D103G, K257R or G319E largely rescues [5,23,35]. Here we use five of those alleles as controls, while testing the impact of expressing three novel alleles: Y105C, N268D and M284K.

**Fig. 2:**
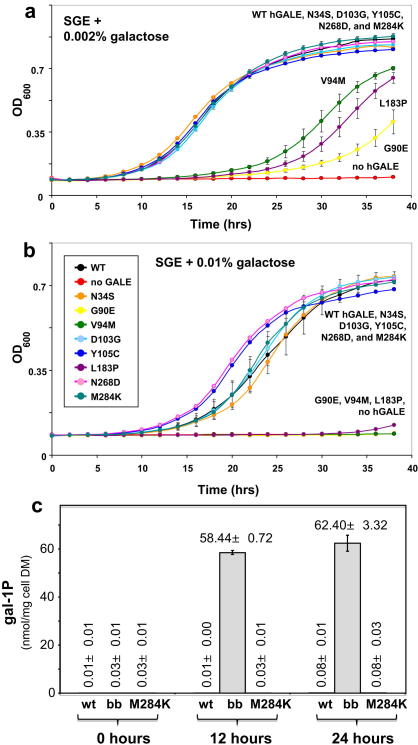
Growth and gal-1P accumulation in *S. cerevisiae* Δ*gal10* expressing wild-type or variant alleles of *hGALE* from centromeric plasmids. Yeast cells were grown at 30 °C in SGE-ura medium supplemented with (a) 0.002% (w/v) or (b) 0.01% (w/v) galactose. Growth was monitored by measurement of the optical density at 600 nm (OD_600_). All strains grew normally in SGE-ura medium without galacose (not shown). (c) Intracellular gal-1P levels in JFy3835 yeast expressing wild-type hGALE (wt), no GALE (bb), or M284K-hGALE (M284K). Cultures grown in SGE-ura medium were supplemented with 0.01% galactose at *t* = 0; samples were harvested for analysis of intracellular gal-1P as described in Materials and Methods at *t* = 0 h, *t* = 12 h, and *t* = 24 h. Values plotted represent mean ± SEM, *n* = 3. The level of gal-1P detected in “bb” samples at 12 h and 24 h was statistically distinct from the corresponding values detected in wild-type or M284K samples by two-tailed Student *t*-test, *p* < 0.001. Note no gal-1P was detected at any time points in control cultures not supplemented with galactose (data not shown).

According to the Western blot and *in vitro* GALE activity assays (Fig. 1), N268D-hGALE is expressed well and retains >60% residual activity, Y105C-hGALE is expressed well and retains >10% residual activity, and M284K-hGALE accumulates in cells only to very low levels and demonstrates no detectable GALE activity. From prior studies [22], we would have predicted that otherwise GALE-deficient yeast expressing N268D-hGALE or Y105C-hGALE would grow well despite the presence of 0.002% or even 0.01% galactose in their medium, and that is what we observed (Fig. 2). None of the variants affected growth in SGE-ura medium lacking galactose (data not shown). We would also have predicted that otherwise GALE-deficient yeast expressing M284K-hGALE would not grow well in the presence of 0.002% or 0.01% galactose, but that is not what we observed (Fig. 2a,b). Instead, the yeast expressing M284K-hGALE grew as well in the presence of galactose as did strains expressing wild-type or mildly-affected alleles of *hGALE*. Further, as predicted from their growth, yeast expressing M284K-hGALE as their only GALE demonstrated no apparent accumulation of gal-1P despite exposure to 0.01% galactose (Fig. 2c), further implicating the M284K allele as functional *in vivo*. In short, M284K-hGALE did not behave according to the anticipated relationship between activity *in vitro* and *in vivo*. To explore the basis for this unexpected result, we pursued detailed biochemical studies of recombinant M284K-hGALE.

### 3.3 Biochemical properties of recombinant M284K-hGALE

**Fig. 3:**
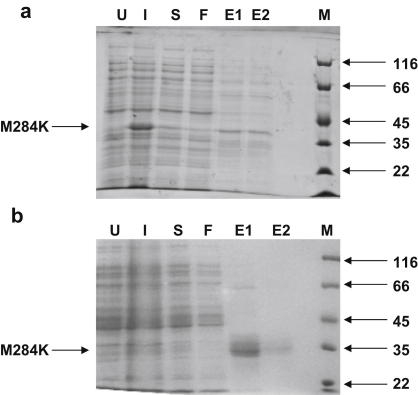
Expression and purification of M284K-hGALE from *E. coli*. Expression levels in cells grown at 37 °C (a) and at 24 °C (b) were monitored by SDS-PAGE (10%), stained with Coomassie blue. Greater amounts of the protein could be produced when the cells were grown at 24 °C; however, even at this temperature some degradation is evident. In both (a) and (b) U, extract from cells immediately prior to induction; I, extract from cells at the end of the period of induction prior to harvesting; S, the supernatant remaining after sonication and centrifugation of the cells; F, the material passing through the column; E1 and E2, the first and second elutions respectively; M, molecular mass markers (kDa).

Given the unusual behaviour of the M284K variant in the yeast model, we decided to investigate its properties further. Prior studies had shown that the wild-type and many variant forms of hGALE could be expressed in, and purified from, *E. coli* cells grown at 37 °C [19,20]. This was not the case with the M284K variant and virtually no protein was produced using this protocol (Fig. 3a). However, when the cells were grown at 24 °C, a detectable amount of hGALE was purified (Fig. 3b). Although the M284K-hGALE protein appeared to be expressed at similar levels to its wild-type counterpart, it tended to precipitate on dialysis, reducing overall yields.

**Fig. 4:**
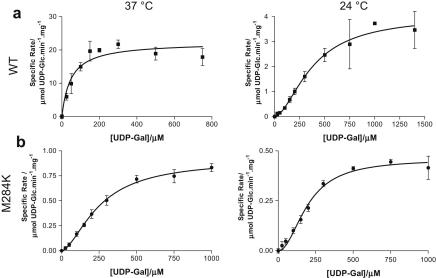
The M284K variant has lower enzymatic activity than does wild-type hGALE. The steady state enzyme kinetics for (a) 5 nM wild-type and (b) 1000 nM M284K variant GALE are shown. Each point represents the mean of three independent determinations of the rate and the error bars represent the standard deviations of these means. In both cases, reactions were carried out at 37 °C and 24 °C in the presence of 1.2 μM coupling enzyme (UDP-glucose dehydrogenase) and 10 mM NAD^+^ at pH 8.8.

The M284K variant demonstrated altered kinetic properties compared to recombinant wild-type hGALE. The *V*_max_ was decreased approximately 24-fold at 37 °C (Fig. 4 and Table 1). Similar effects were also observed at 24 °C (Table 1). Interestingly, the rate of reaction catalysed by the variant exhibited a sigmoidal dependence on substrate concentration at both temperatures. The wild-type enzyme only exhibited this behaviour at the lower temperature (Fig. 4 and Table 1).

**Fig. 5:**
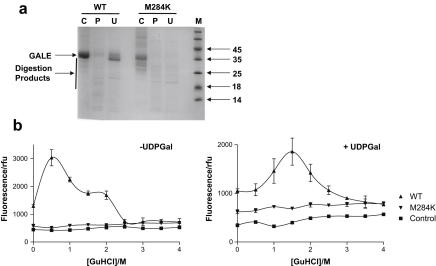
Limited proteolysis of wild-type hGALE and the M284K variant showed greater degradation of the M284K variant compared to the wild-type protein. UDP-galactose partially protected the wild-type protein, but not the M284K variant from proteolysis. C, control, undigested protein (10 μM); P, protein digested with 600 nM trypsin; U, protein digested with 600 nM trypsin in the presence of 1 mM UDP-galactose. (b) Guanidine hydrochloride denaturation of the wild-type protein followed by ANS fluorescence. In the absence of UDP-galactose (left), a peak occurred at approximately 0.8 M GuHCl. This peak shifted to approximately 1.6 M GuHCl in the presence of 50 μM UDP-galactose (right). No such effects were seen with the M284K variant. The control data represent the fluorescence of ANS in increasing concentrations of GuHCl in the absence of protein. Each point represents the mean of three determinations and the error bars represent the standard deviations of these means.

The variant was also much less stable than the wild-type protein. Limited proteolysis assays showed that this variant was almost completely digested under conditions where the wild-type protein was cleaved into discrete fragments (Fig. 5a). In contrast to the wild-type protein, the substrate (UDP-galactose) did not protect M284K-hGALE from this digestion (Fig. 5a). The structural stability of M284K was further investigated using the hydrophobic fluorophore 1-anilinonaphthalene-8-sulphonic acid (ANS). This compound binds to exposed hydrophobic patches on proteins; this binding results in an increase in ANS fluorescence. Typically ANS binds weakly to fully folded, globular proteins which have their hydrophobic residues largely in the interior. However, as unfolding begins (for example, in response to a denaturing agent) hydrophobic patches are exposed. As the process continues, and the partially folded protein is converted to a random coil, fully unfolded polypeptide, these hydrophobic regions are disrupted and ANS binding often decreases [36]. The compound has been shown to bind at, or near, the active site of *E. coli* GALE [37]. Wild-type hGALE exhibited a typical unfolding profile in the presence of increasing concentrations of guanidine hydrochloride (GuHCl) with maximum ANS binding at approximately 0.8 M GuHCl. Addition of the substrate, UDP-galactose (50 μM), stabilised the wild-type protein and shifted the concentration of GuHCl required for maximum binding of ANS to approximately 1.6 M (Fig. 5b,c). In contrast, the M284K variant showed no binding to ANS in the presence or absence of substrate over the range of GuHCl concentrations tested (0–4M). Loss of interaction with ANS upon denaturation has also been demonstrated with *Kluyveromyces fragilis* GALE [38].

## 4 Discussion

We report here a combination of *in vitro* and *in vivo* studies of eight variant alleles of human *GALE* – five patient-derived and three resulting from a laboratory-based mutagenesis screen. Our results demonstrate that seven of the eight alleles followed the previously-predicted relationship between *in vitro* and *in vivo* function, but one, M284K, did not. That two of three “artificial” *hGALE* alleles demonstrated the same apparent relationship between *in vitro* and *in vivo* function as was seen for the five patient-derived alleles suggests that similar exceptions might also exist among naturally-occurring alleles. Naturally-occurring alleles that function well *in vivo* are unlikely to come to clinical attention, however, and therefore may not be recognised.

Kinetic and stability studies of recombinant M284K-hGALE revealed a protein compromised in both parameters. The low enzymatic activity, tendency to precipitate, high susceptibility to proteolysis and lack of ANS binding of M284K-hGALE all suggested that this variant is largely unfolded in solution *in vitro*. However, its ability to complement a Δ*gal10* yeast strain, demonstrates that it can function *in vivo*. Thus, for this variant, *in vitro* analysis is a poor predictor of *in vivo* function, and one must conclude that the protein instability may be limited to *in vitro* contexts. Potentially critical differences between the *in vitro* and *in vivo* environments are innumerable, and range from issues such as net protein concentration to the presence of chaperones *in vivo* (which may help maintain a structurally unstable variant in a folded, soluble, active form) and even the possibility that the Leloir pathway enzymes may be organised into a supra-molecular complex (or metabolon) [39]. Interactions with the other proteins, including other Leloir pathway enzymes, in theory might help to reinforce and stabilise the structure of GALE *in vivo*. Of course, the preparation of a cell lysate, especially from microbes with cell walls, such as bacteria or yeasts, would likely disrupt these interactions, thereby destabilising the M284-hGALE protein and leading to the Western blot and enzyme activity results presented here. Wild-type hGALE would similarly lose its potentially stabilising interactions or influences, but it might be sufficiently resilient to withstand the loss. While the nature of the *in vivo* “GALE stabilizing factor or factors” remains unclear, the results reported here suggest that these factors are likely to be conserved between yeast and humans.

It would be interesting to extend our experiments to express M284K-hGALE and other alleles of human GALE in the null-background mammalian cell line, ldlD, as has been described previously for wild-type human GALE and *E. coli* GALE [40], as well as a set of patient alleles [21]. Of note, even in this mammalian expression system aggregation of some disease-associated variants of GALE has been observed [21]. For the M284K variant it would be especially interesting to quantify synthesis level, stability, localisation, and possible aggregation, to see if higher levels of soluble, active M284K-hGALE protein might be detected in a mammalian system. Such experiments would also permit extracts to be prepared and the activity of the protein derived from this source to be assessed *in vitro*.

**Fig. 6:**
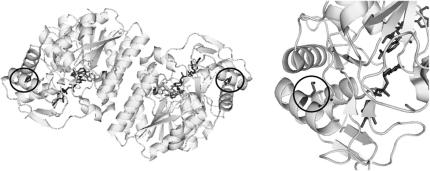
Met-284 is located towards the surface of the protein, away from the active site and NAD^+^ binding site. The left hand figure shows the location of this residue (circled) in both subunits of the hGALE homodimer. The right hand view shows a close-up of the residue in one subunit. The side chain of Met-284 (circled) points towards the centre of a bundle of three α-helices. Substitution of this residue for lysine will insert an unpaired positive charge into this bundle. The figures were made in PyMol (www.pymol.org) using PDB entry 1EK6 [41].

The existence of a high resolution crystal structure for hGALE [2,41] enables some speculation about the structural consequences of altering methionine to lysine at position 284. The residue is located far from the dimer interface and is part of an α-helix on the surface of the protein (Fig. 6). The side chain points towards the centre of a triple helix bundle and the change to lysine, with the consequent introduction of an unpaired positive charge, is likely to disrupt the packing of this helix bundle. That this substitution has such profound consequences for the stability of the whole protein, suggests that this α-helical bundle may be a key structure in the folding pathway of the protein. Furthermore, if interactions with other proteins do help stabilise hGALE *in vivo*, then they are likely to occur close to this region.

The observation of non-Michaelis–Menten behaviour by the M284K variant (and the wild-type enzyme at reduced temperature) is interesting. Although this behaviour by the wild-type human enzyme at 37 °C has not been previously reported, there is considerable evidence for allostery in GALE isolated from the yeast *K. fragilis* [42–45]. At present, the consequences of this allostery in the human enzyme are not clear. That no (or limited) allostery is observed in the isolated wild-type enzyme at 37 °C suggests that there may be only specific circumstances *in vivo* under which it is more pronounced. For example, it is possible that, like in many other metabolic enzymes, hGALE’s activity can be regulated by small molecule effectors. It is also possible that, like in *K. fragilis*, the allostery is more pronounced in the reverse reaction.

The results reported here demonstrate that biochemical tests on hGALE *in vitro* will not always predict behaviour *in vivo*. For the majority of variants investigated, there was a clear correlation between their ability to complement galactose sensitivity of the Δ*gal10* yeast strain and the enzymatic activity in cell extracts. This was not the case with the M284K variant which was functional in the context of a living yeast cell, but compromised as an isolated protein, or in a soluble cell-free lysate. This finding emphasises the need to investigate the properties of newly discovered hGALE variants both *in vitro* and in model *in vivo* systems.

## Figures and Tables

**Table 1:** Kinetic constants of recombinant wild-type and M284K variant hGALE enzymes.

Protein	Temperature, °C	*K*_m_ or *K*_0.5_, μM	*V*_max_, mmol UDP-Glc min^−1^ mg^−1^	*h*
hGALE	37	48 ± 16	22 ± 2	n/a
hGALE	24	380 ± 48	4.0 ± 0.3	1.7 ± 0.2
M284K-hGALE	37	250 ± 18	0.91 ± 0.04	1.7 ± 0.1
M284K-hGALE	24	200 ± 16	0.46 ± 0.02	2.0 ± 0.3
