# Supplementary material for: In vivo and in vitro function of human UDP-galactose 4′-epimerase variants
Source: Biochimie. 2011 Oct;93(10):1747–54. doi: 10.1016/j.biochi.2011.06.009 (PMC3168732; doi:10.1016/j.biochi.2011.06.009)
Supplement: Supplementary file 1 [file mmc1.ppt]

## Slide 1
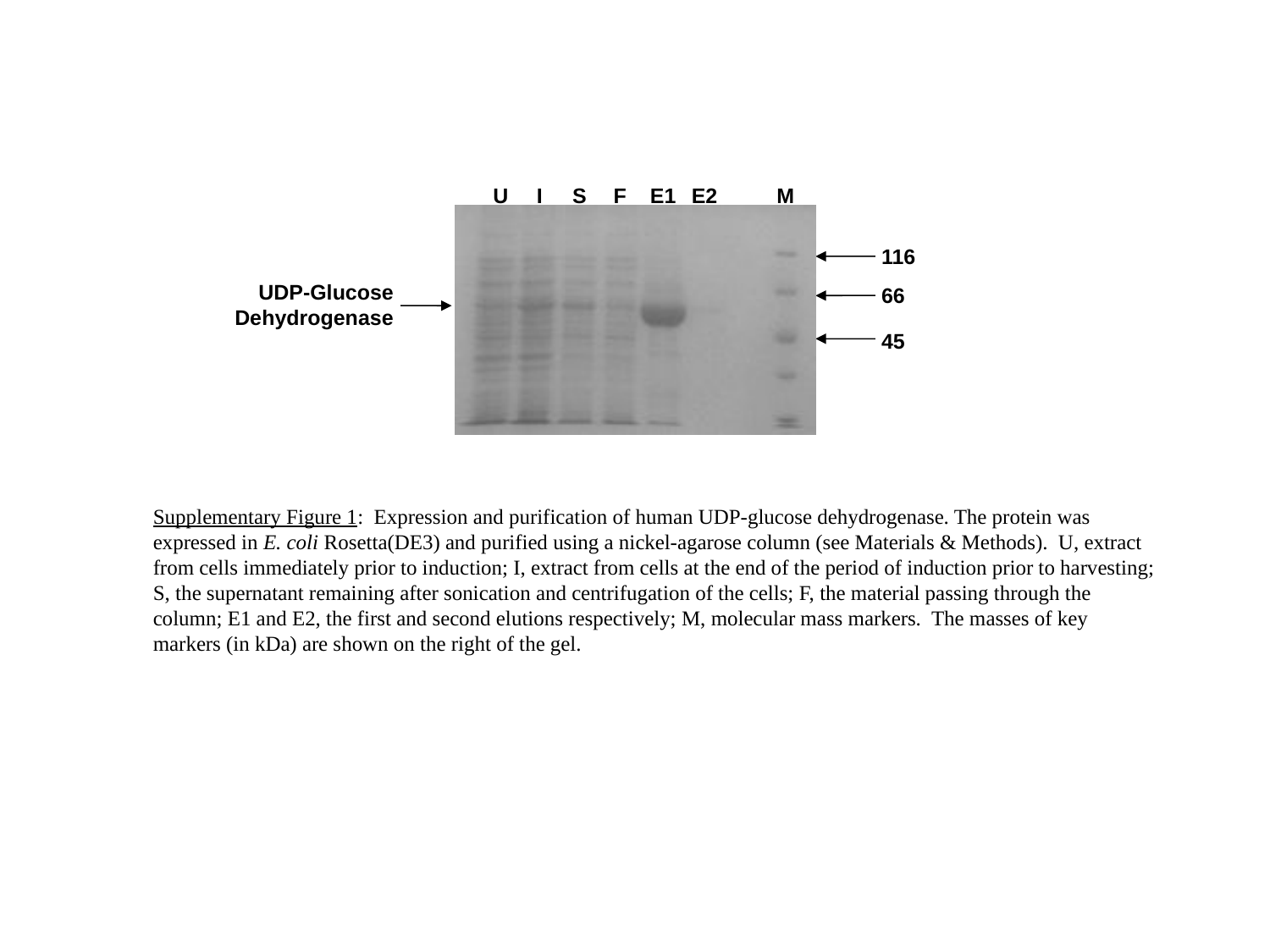

U
 I
 S
 F
 E1
 E2
 M
116
UDP-Glucose
Dehydrogenase
66
45
Supplementary Figure 1: Expression and purification of human UDP-glucose dehydrogenase. The protein was expressed in E. coli Rosetta(DE3) and purified using a nickel-agarose column (see Materials & Methods). U, extract from cells immediately prior to induction; I, extract from cells at the end of the period of induction prior to harvesting; S, the supernatant remaining after sonication and centrifugation of the cells; F, the material passing through the column; E1 and E2, the first and second elutions respectively; M, molecular mass markers. The masses of key markers (in kDa) are shown on the right of the gel.
