# Supplementary material for: In vivo and in vitro function of human UDP-galactose 4′-epimerase variants
Source: Biochimie. 2011 Oct;93(10):1747–54. doi: 10.1016/j.biochi.2011.06.009 (PMC3168732; doi:10.1016/j.biochi.2011.06.009)
Supplement: Supplementary file 2 [file mmc2.doc]

**Supplemental Table S1:** Plasmids used in this study.

___________________________________________________________________

**Plasmid hGALE**

**Number Type Name Allele**

JF2812 centromeric pMM33 (no GALE) none

JF2899 centromeric pMM33.hGALE Wild-type

JF3994 centromeric pMM33.N34S N34S

JF2901 centromeric pMM33.G90E G90E

JF3991 centromeric pMM33.V94M V94M

JF3988 centromeric pMM33.D103G D103G

JF4015 centromeric pMM33.Y105C Y105C*

JF4013 centromeric pMM33.L183P L183P

JF4017 centromeric pMM33.N268D N268D*

JF4044 centromeric pMM33.M284K M284K*

JF2810 high copy (2µ) pMM195 (no GALE) none

JF4132 high copy (2µ) pMM195.M284K M284K*

* *hGALE* alleles not derived from patient mutations

**Supplemental Table S2.** Yeast strains used in this study

_____________________________________________________________________

**JFy hGALE**

**Number Plasmid Allele**

JFy4156, 4157, 4158 JF2812 (CEN) none

JFy4137, 4138, 4139 JF2899 (CEN) Wild-type

JFy4125, 4126, 4127 JF3994 (CEN) N34S

JFy4140, 4141, 4142 JF2901 (CEN) G90E

JFy4128, 4129, 4130 JF3991 (CEN) V94M

JFy4131, 4132, 4133 JF3988 (CEN) D103G

JFy4159, 4160, 4161 JF4015 (CEN) Y105C*

JFy4173, 4174, 4175 JF4013 (CEN) L183P

JFy4162, 4163, 4164 JF4017 (CEN) N268D*

JFy4226, 4227, 4228 JF4044 (CEN) M284K*

JFy4364, 4365, 4366 JF2810 (2μ) none

JFy4352, 4353, 4354 JF4132 (2μ) M284K*

* hGALE alleles not derived from patient mutations
